# Interferencia del metamizol en la determinación de la concentración de creatinina sérica

**DOI:** 10.1515/almed-2024-0065

**Published:** 2024-06-24

**Authors:** María-José Castro-Castro, Isabel Cachón-Suárez, Andrea Maestre-Fernández, Rosa Navarro-Badal

**Affiliations:** Laboratory Medicine Department, 16383 Hospital Universitario de Bellvitge, Barcelona, España; Biochemistry and Physiology Department, 16724 Universitat de Barcelona, Barcelona, España

**Keywords:** interferencia del metamizol, método enzimático de creatinina

## Abstract

**Objetivos:**

Existen escasos estudios sobre la interferencia del metamizol en la determinación de la concentración de creatinina mediante el método enzimático. En algunos estudios, se ha identificado a la molécula dipirona como la responsable de interferir en la última reacción de la secuencia del método enzimático, a causa de su similitud con la molécula 4 aminofenazona. El objetivo del presente estudio es analizar de qué modo la presencia de metamizol interfiere en la determinación de la concentración de creatinina sérica cuando se utiliza el método enzimático.

**Métodos:**

Se realizó un estudio de interferencia de la determinación de la creatinina aplicando dos procedimientos de medición (enzimático y método de Jaffé), añadiendo diferentes concentraciones de metamizol a un *pool* de 30 muestras de suero de pacientes.

**Resultados:**

El estudio de interferencia reveló que, al añadir concentraciones crecientes de metamizol, se produce una disminución en los resultados de las concentraciones de creatinina sérica, cuando estas se determinan con el método enzimático.

**Conclusiones:**

En el método enzimático, la presencia de metamizol interfiere en la medición de la concentración de creatinina sérica, esta interferencia no se observa en el método de Jaffé.

## Introducción

La creatinina sérica es uno de los componentes más frecuentemente analizados en los laboratorios de química clínica de todo el mundo [1]. Hasta hace poco, el método más utilizado para medir la creatinina era el método de Jaffé, que consiste en inducir la reacción de la creatinina con el ácido pícrico en una solución alcalina. Aunque se trata de un método sencillo y económico, la reacción de Jaffé es inespecífica, ya que el picrato alcalino puede reaccionar con cualquier compuesto que contenga un grupo metileno. Aunque se han introducido algunas modificaciones cinéticas para reducir la interferencia en el método de Jaffé, estas no han logrado resolver completamente sus limitaciones en términos de especifidad [2, 3]. El método enzimático para la determinación de la creatinina se describió por primera vez en 1937, siendo inherentemente más específica, dada la selectividad de las enzimas [2]. Aunque las pruebas de Jaffé son más susceptibles a las sustancias interferentes que el método enzimático, tanto en la frecuencia como en el grado de interferencia, las pruebas enzimáticas tampoco son inmunes a la inespecifidad [4]. Así, en las especificaciones de uno de los reactivos empleados en el método enzimático actualmente comercializados por Roche Diagnostics, el fabricante declara interferencias con rifampicina, levodopa, dobesilato cálcico, metildopa, N-etilglicina, DL-prolina, 2-fenil-1,3-indandio (fenindiona), dicinona (etamsilato) y metamizol, entre otros [5].

Aunque se desconoce la frecuencia de los errores causados por interferencias analíticas, de acuerdo con la literatura existente, los errores producidos durante la fase analítica representan entre el 7 y el 13 % de la totalidad de los errores [6]. Existe escasa literatura sobre la interferencia del metamizol descrita por Roche Diagnostics en la determinación de la concentración de creatinina mediante el método enzimático. Según Bagnoud y Reymond, el metabolito metil-amino-antipirina parece ser el responsable de la interferencia. Dicho metabolito es el principio activo, ya que el metamizol es un profármaco [7].

En algunos estudios, se han descrito algunos mecanismos de interferencia del metamizol u otros fármacos con métodos analíticos [8, 9]. De este modo, la molécula dipirona podría ser la responsable de interferir en la última reacción de la secuencia del método enzimático, debido a su similitud con la molécula 4-aminofenazona.

Estudios previos *in vitro* muestran que el metamizol interfiere con el método de Jaffé y el método enzimático a diferentes concentraciones de metamizol (concentraciones subterapéuticas, terapéuticas, o tóxicas) [10]. Un estudio realizado por Bojko L. y col. reveló que la administración intravenosa de metamizol interfería en la determinación de la concentración de creatinina mediante el método enzimático [11]. Sin embargo, otro estudio descartó que el metamizol interfiriera con el método enzimático de Randox para la determinación de la concentración de creatinina [12].

Así mismo, se han descrito otras interferencias farmacológicas, la totalidad de las cuales afectan a la reacción de Trinder (la responsable del último paso de la reacción para la determinación de la creatinina con el método enzimático) [9].

Debido a su uso extendido como analgésico, alrededor del 21 % de los pacientes hospitalizados del Hospital Universitario de Bellvitge (L’Hospitalet de Llobregat, Barcelona, Spain) recibieron tratamiento intravenoso con metamizol, frente al 18 % al que se les administró por vía oral. De este modo, es necesario determinar en que medida este fármaco interfiere en la determinación de magnitudes bioquímicas en el laboratorio clínico.

El objetivo del presente estudio es analizar de qué modo interfiere la presencia de metamizol en la medición de la concentración de creatinina sérica a través del método enzimático.

## Materiales y métodos

La concentración de creatinina se midió en un analizador Cobas c702 (Roche Diagnostics, Rotkreuz, Suiza). Se evaluó la calidad analítica de los dos procedimientos, el método enzimático y el método de Jaffé, a la hora de determinar la concentración de creatinina sérica. La media del coeficiente de variación analítico y el error sistemático durante el estudio de los dos métodos, se calculó aplicando dos niveles de control de calidad interno: los niveles 1 y 3 de los materiales de control Liquid Assayed Multiqual (referencias 694 y 696, lote 45,890), fabricados por Bio-Rad (Madrid, España), que se procesaron durante 16 días. Se calcularon el coeficiente de variación y el error sistemático, asumiendo como valor verdadero la media de los valores del grupo con el mismo método de medida en los datos acumulados del Programa de Garantía de Calidad Externa de Bio-Rad.

Realizamos un estudio de interferencia con la determinación de la creatinina aplicando los dos métodos de medición (enzimático y de Jaffé), añadiendo diferentes concentraciones de metamizol a un *pool* de suero, con el fin de obtener una mezcla de 30 muestras de suero de pacientes, con una concentración media de 126 μmol/L. Dicha concentración no dista mucho del valor de referencia para la población general. En el intervalo de concentraciones de metamizol, se incluyeron las concentraciones terapéuticas máximas (C_max_) documentadas en algunos estudios farmacocinéticos para su administración endovenosa: 0,03 g/L [13], 0,05–0,1 g/L [14].

Para preparar diferentes diluciones de metamizol, se utilizaron viales de metamizol de 2 g/5 mL (400 g/L), obteniendo soluciones de 100 g/L, 20 g/L, 10 g/L, 5 g/L, 2,5 g/L, 1,25 g/L, 0,63 g/L, 0,31 g/L y 0,16 g/L añadiendo cloruro sódico. Se añadieron 20 µL de cada solución de metamizol a diferentes alícuotas del *pool* de suero, de 180 µL cada una. Además, como control negativo, se añadieron 20 µL de cloruro sódico a 180 µL del *pool* de suero. Se realizó la prueba de Wilcoxon usando las medianas para comparar los resultados obtenidos con los dos procedimientos de medición. Un valor p <0,05 se consideró estadísticamente significativo.

Se calculó la diferencia en el porcentaje de concentración de creatinina sérica de cada dilución con respecto a las muestras de control. A continuación, se compararon dichas diferencias con el error total permitido establecido en nuestro laboratorio, basados en el estado del arte (20 %) [15].

Se almacenaron a −20 °C seis muestras de suero de pacientes con sospecha de contaminación por la administración intravenosa de metamizol. La sospecha de interferencia se fundamentó en que, durante el proceso de validación de resultados, se comprobó que el resultado en estas muestras era muy diferente del resultado anterior en el mismo paciente. Así mismo, el sistema de límites de cambio (*deltacheck*) aplicado en el laboratorio clínico alertó sobre la necesidad de revisar los resultados (25 % de disminución en el límite de cambio y 40 % de aumento en el límite de cambio).

Estas muestras pertenecían a pacientes ingresados en distintas unidades hospitalarias (urgencias, medicina intensiva, cuidados paliativos, cardiología y gastroenterología). Las muestras se descongelaron y procesaron empleando los dos procedimientos de medición (enzimático y de Jaffé).

Los datos personales de los pacientes obtenidos para los fines del presente estudio se procesaron de conformidad con el Reglamento (UE) 2016/679 del Parlamento Europeo sobre Protección de Datos. Así mismo, el estudio se realizó con ajuste a los principios éticos para la investigación médica en seres humanos de la Declaración de Helsinki de la World Medical Association. El Comité Ético para la Investigación del Hospital Universitario de Bellvitge aprobó la publicación del presente artículo (número de referencia PR240/22).

Los análisis estadísticos se realizaron con el programa SPSS versión 17.0 y con Microsoft Excel 2010.

## Resultados

Se calcularon el coeficiente de variación y el error sistemático, observando que los métodos enzimáticos y de Jaffé se ajustaban a las especificaciones de calidad establecidas por nuestro laboratorio, en las que se establece un 7.4 % de imprecisión y un 8.8 % de error sistemático. De este modo, nos aseguramos de que ambos métodos cumplieran los requisitos de calidad establecidos en nuestro laboratorio.

**Figura 1:**
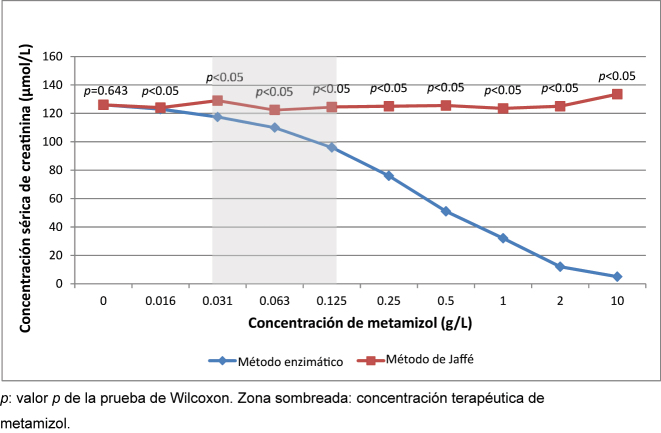
Gráfica con las concentraciones de creatinina sérica de un *pool* de muestras de suero de pacientes, determinadas mediante dos métodos distintos (enzimático y de Jaffé) con la adición de diferentes concentraciones de metamizol. p, valor p de la prueba de Wilcoxon. Zona sombreada, concentración terapéutica de metamizol.

En la Figura 1, se muestra una representación gráfica de las concentraciones de creatinina sérica en el *pool* de suero aplicando dos procedimientos de medición (enzimático y de Jaffé) con diferentes concentraciones de metamizol. También se indica el valor p para la prueba de Wicoxon. La zona sombreada representa la concentración terapéutica de metamizol empleada para la concentración intravenosa. El gráfico muestra un descenso de la concentración de creatinina con el método enzimático cuando aumenta la concentración de metamizol, lo que contrasta con el método de Jaffé, donde dicha reducción no se produce.

Además, en la Tabla 1 se muestran las medianas de concentración de creatinina sérica con el rango intercuartílico y las diferencias porcentuales de cada dilución con respecto a las muestras de control. La zona sombreada indica la concentración terapéutica de metamizol para la concentración intravenosa. Cuando la concentración de metamizol aumenta, se produce un descenso en la concentración de creatinina sérica, así como un elevado porcentaje de variación con respecto a la muestra en la que no se ha añadido metamizol, como se observa en el método enzimático. Cabe señalar que dicha variación no se observa en el método de Jaffé.

**Tabla 1:** Comparación de las medianas de concentración de creatinina en el *pool* de suero mediante los métodos enzimático y de Jaffé, añadiendo concentraciones crecientes de metamizol.

Concentración de metamizol, g/L	Concentración de creatinina con el método enzimático Mediana (RIC), µmol/L (%)	Concentración de creatinina con el método de Jaffe Mediana (RIC), µmol/L (%)
0	126 (123,130)	126 (124,130)
	(0,0 %)	(0,0 %)
0,016	123 (123,130)	124 (122,127)
	(2,4 %)	(1,6 %)
0,031	118 (110,121)	129 (127,132)
	(6,7 %)	(2,4 %)
0,063	110 (108,113)	123 (122,126)
	(12,7 %)	(2,8 %)
0,125	96 (93,100)	125 (123,128)
	(23,8 %)	(1,2 %)
0,25	76 (74,78)	125 (123,129)
	(39,7 %)	(0,8 %)
0,5	51 (49,52)	126 (124,130)
	(59,5 %)	(0,4 %)
1	33 (32,34)	124 (123,126)
	(74,6 %)	(2,0 %)
2	12 (11,12)	125 (123–126)
	(90,5 %)	(0,8 %)
10	5 (5,6)	134 (133,135)
	(96 %)	(6,0 %)

Zona sombreada, concentración terapéutica de metamizol; %, porcentaje de variación con respecto a la ausencia de metamizol; RIC, rango intercuartílico (Q1, Q3).

En la Tabla 2 se muestran los resultados de concentración de creatinina determinada con los dos métodos, en muestras de suero en las que se sospechaba que había contaminación a causa de la administración intravenosa de metamizol. En algunas muestras procesadas (muestras 1, 3, 5 y 6), se observa una variación significativa en la concentración de creatinina entre el método enzimático y el método de Jaffé. La concentración medida con el método enzimático se mantuvo persistentemente más baja que la obtenida con el método de Jaffé.

**Tabla 2:** Concentraciones de creatinina en muestras de suero ante la sospecha de contaminación por la administración intravenosa de metamizol, obtenidas con el método enzimático y de Jaffé.

Muestra de suero	Concentración de creatinina con el método enzimático, µmol/L	Concentración de creatinina con el método de Jaffé, µmol/L
Muestra 1	15	63
Muestra 2	42	41
Muestra 3	8	93
Muestra 4	27	28
Muestra 5	3	110
Muestra 6	2	141

## Discusión

El gráfico (Figura 1) en el que se comparan los resultados de la concentración de creatinina sérica obtenidos mediante el método enzimático y de Jaffé, muestra una tendencia distinta: en el método enzimático, cuando la concentración de metamizol aumenta, el efecto de interferencia es más acusado. Por el contrario, el método de Jaffé no muestra dicha interferencia a mayores concentraciones de metamizol.

Este hallazgo indica claramente que la presencia de metamizol provoca una interferencia en el método enzimático de determinación de la creatinina sérica, lo que no ocurre con el método de Jaffé, en el que no parece producirse ninguna interferencia. De hecho, esta interferencia podría hacer que el resultado de concentración de creatinina quede por debajo del intervalo de medida.

Además, la prueba de Wilcoxon reveló diferencias significativas (p<0,05) en todas las diluciones, excepto en el control (ausencia de metamizol), lo que confirmó la presencia de diferencias considerables entre los dos métodos. Este hallazgo demuestra inequívocamente que el método enzimático es sensible a la interferencia causada por el metamizol, cuando sus concentraciones exceden los 0,016 g/L en la muestra, no viéndose afectado sin embargo el método de Jaffé.

Según los resultados obtenidos, cuando comparamos los porcentajes de creatinina sérica con el error total admisible, las muestras con concentraciones de metamizol de hasta 0,062 g/L fueron analíticamente relevantes.

De acuerdo con los estudios farmacocinéticos [13], las concentraciones máximas de metamizol en plasma tras su administración por vía intravenosa oscilan entre los 0,03 y los 0,015 g/L al cabo de una hora, y 0,15 g/L transcurridas 2 horas desde la administración oral (dosis endovenosas y orales de 1,000 mg). Es posible que las concentraciones séricas elevadas pudieran ser causadas por la punción venosa directa, que podría provocar contaminación debido a la administración intravenosa. Una limitación del presente estudio es la ausencia de determinaciones seriadas tras la administración de metamizol.

En la Tabla 2, se muestra una discrepancia significativa en las concentraciones de creatinina entre las muestras 1, 3, 5, y 6 medidas con el método enzimático y de Jaffé. Esta variación sugiere que el metamizol puede interferir con el método enzimático, lo que podría influir en los procesos asociados a la reacción de Trinder [8, 9].

Nuestros resultados coinciden con estudios específicos [10, 11], que documentan la interferencia del metamizol en la determinación de la creatinina con el método enzimático, aunque los resultados en relación al método de Jaffé no son consistentes. Mientras que Luna-Zaizar y col. observaron una interferencia positiva, nuestro estudio no reveló ninguna interferencia en el método de Jaffé.

Otra limitación del estudio, es que se excluyeron del análisis los principales metabolitos del metamizol, debido a limitaciones en el intervalo de medida, lo que es una importante limitación de nuestro estudio. La capacidad de detectar muestras susceptibles de interferencia mejoraría si se pudieran determinar las concentraciones de estos metabolitos. Sin embargo, nuestros resultados indican que, en los pacientes a los que se administra metamizol por vía intravenosa, se pueden obtener concentraciones de creatinina falsamente bajas.

En nuestro estudio, no pudimos analizar la interferencia a diferentes concentraciones de creatinina, por lo que son necesarios más estudios que no presenten esta limitación.

Para los laboratorios clínicos, puede resultar difícil identificar en la práctica las muestras susceptibles a esta interferencia. Normalmente, la sospecha surge durante la validación de los resultados, especialmente, cuando se observa una reducción notable de la concentración con respecto a los resultados anteriores del mismo paciente. De este modo, resulta esencial contactar con el servicio de extracciones para averiguar si se ha administrado recientemente metamizol intravenoso. Estos pasos son básicos a la hora de identificar la interferencia y, en caso de que se detectara, resulta necesario obtener una nueva muestra para evitar obtener resultados inexactos de creatinina sérica.

Aunque mantener el método de Jaffé y el enzimático podría ser inviable para muchos laboratorios clínicos, el método de Jaffé, que no está sujeto a interferencias del metamizol, podría servir como herramienta para la confirmación de una posible interferencia. No obstante, en caso de sospecha de interferencia, resultaría más práctico solicitar una nueva muestra del paciente, sumado a la mejora de los procesos preanalíticos de obtención de muestras, con el fin de mitigar el riesgo de contaminación intravenosa.

Sin embargo, actualmente, aún se desconoce la dosis exacta de metamizol intravenoso a partir de la cual se produce la interferencia, lo que evidencia la necesidad de realizar estudios *in vivo* con obtención de muestras seriadas tras la administración de metamizol. El laboratorio debería difundir directrices entre el personal de enfermería del hospital, en las que se haga hincapié en la importancia de extraer la sangre antes de administrar metamizol.

En conclusión, cuando se emplea el método enzimático, el metamizol podría interferir en la determinación de la creatinina sérica, lo que no ocurre con el método de Jaffé. Para determinar si la concentración de creatinina se ha visto reducida por error al emplear el método enzimático, podría ser necesario reprocesar la muestra utilizando el método de Jaffé. La interferencia observada con el metamizol en la determinación de la creatinina sérica probablemente se debe a procesos específicos del método enzimático, en contraposición al método de Jaffé, que no es susceptible de dichas interferencias.
